# Connected and extracted: Understanding how centrality in the global wheat supply chain affects global hunger using a network approach

**DOI:** 10.1371/journal.pone.0269891

**Published:** 2022-06-15

**Authors:** Subhashni Raj, Catherine Brinkley, John Ulimwengu

**Affiliations:** 1 Department of Human, Ecology, University of California, Davis, CA, United States of America; 2 International Food Policy Research Institute, Washington, DC, United States of America; Szechenyi Istvan University: Szechenyi Istvan Egyetem, HUNGARY

## Abstract

While global food trade has allowed countries to buffer against domestic food production shortfalls and gain access to larger markets, engaging in trade has also opened economies up to shocks and increased extraction of food resources. With this research, we analyze how the global grain network influences country-level nourishment, while controlling for per capita land and food production. First, we model the trade network structure of the global wheat supply chain to measure the centrality or positionality of countries. We use spatial regression analysis to assess the impact of trade networks, volume, purchasing power, production capacity and geography on undernourishment. We find that the six countries most central to the global grain trade by betweenness and eigenvector centralities account for more than half of all wheat exports globally by volume. The centrality of these countries as opposed to volume of wheat produced or traded, determines their influence in the wheat supply chain network. The parametric component of our analysis confirms that trade, and centrality have significant implications for national levels of nourishment. Our findings suggest that for countries with low purchasing power, increasing centrality allows improvements in nourishment levels but for countries with very high purchasing power, increasing centrality can increase hunger outcomes. To counteract perturbations and shortfalls such as those being experienced currently in the globalized food system, local and regional governments may consider refocusing on regional and local based food systems.

## Introduction

With this research, we ask how the amount of arable land per capita, purchasing power parity, food production and trade networks influence country-level nourishment. This question is at the heart of debates over sustainability, the global economy, and policies and attitudes towards self-reliance. While global food trade has allowed countries to buffer against domestic food production shortfalls and gain access to larger markets, engaging in trade has also opened economies up to shocks and extraction of food resources [1–4]. Nearly a quarter of all food produced for human consumption is traded globally [5], and while global food supply has increased since the 1960’s, food self-sufficiency has not changed remarkably [6], intertwining trade and hunger outcomes. Despite enough food for human consumption being produced and traded globally [5, 7], an estimated 720 to 811 million people are hungry [8]. The UN Food and Agriculture Organization (FAO) defines hunger as a situation when people are not able to consume sufficient caloric energy on a regular basis to live an active and healthy life, measured as undernourishment [8]. Since 2014, the FAO has noted an uptick in global undernourishment levels which rose 9.5 percent between 2014 and 2019. The global COVID-19 pandemic added another 161 million people to the ranks of undernourished in 2020 [8]. Such statistics point to trade as central in solving hunger while also accelerating the achievement of the United Nations (UN) Sustainable Development Goal on ending hunger [8]. Policy responses range from calls to convert more wildlands to arable land, intensify crop production, or redistribute what is produced by shifting trade policies to redirect exports or emphasize isolation from global markets [9].

Recently, scholars have begun assessing the global food and trade system using network analysis [2, 7, 10–12]. Network analysis of the global food trade shows that the world has become more interconnected over the last thirty years but not anymore volatile [10]. Research shows that the intensification of trade in itself does not erode food system resilience but that resilience is dependent on network structure [13]. Similarly, global wheat and rice commodity networks are evolving to be less vulnerable over time as countries become interconnected with more trading partners [12]. Resilience of global food systems is however undermined when countries are either super exporters or almost entirely trade dependent importers [7, 13]. Modularity also plays an important role in food system resilience [7, 13, 14], a decrease in system modularity was related to increase in critical transitions in the food trade network. Scholars found that increasing imports and import partners in the seafood trade deepened countries’ exposure to shocks [11], but increasing localization, showed a reduced ability for perturbations to propagate [7]. While others found that node centrality increases as the spatial scale decreases, suggesting that certain local food supply sectors are more critical to community food security than countries are to global food supply [15]. Increased delocalization of food systems over the last few decades has contributed to the erosion of food system resilience [11, 13]. Noticeably, as the number of countries sensitive to perturbations in the global food trade has increased between 1986 and 2010, trade-dependent rather than self-sufficient countries are particularly vulnerable to shocks [7]. In turn, the overall global food and trade system is robust against shocks when less central countries (nodes in the network) are impacted in isolation [10, 12, 16]. In sum, while interconnectivity can help dissipate a shock across the global system, some countries are more vulnerable to the effects of a global shock depending on their positioning in the global trade network and purchasing power.

A surge in global food insecurity in the wake of the COVID 19 pandemic due to grain exports restrictions [2, 17] brings urgency to assessing and addressing vulnerability in the global food and trade network. External shocks, like the global pandemic, are expected to create further trade shocks and occur more frequently as we approach mid-century [18–20]. In meeting these pressing policy considerations, researchers are untangling how shocks in trade networks influence food security [1]. Given the reliance of a majority of countries to meet demand for staples and domestic shortfalls through trade [16], the global food system is vulnerable to both self-propagating risks (e.g. trade restrictions) and global systemic risk (e.g. climate change) [10–12, 16, 20]. As sensitivity to perturbations has increased over time, so has food insecurity [7]. Poorer and developing countries absorb more of the trade shock as interconnectivity increases [1, 16, 21]. Such inequitable impacts of trade on developing countries has implications for nourishment and hunger in places already struggling to secure a consistent food supply [21, 22].

While these studies are helpful in understanding the network properties that infer resilience to global models of food trade, there is still limited understanding of who benefits and who loses from the global food trade and the differential effect of food trade on undernourishment. Research does indicate that trade can facilitate access to food especially in poorer countries and improve food and nourishment indicators [6, 23, 24]. Though scholars also find that trade does not improve nutrient adequacy of low/lower-middle income countries substantively, and that on average low/lower-middle income countries imported 70% less foods to meet adequacy needs than upper-middle/high income countries [25]. Evidence also suggests that the growth experienced by western countries was in large part due to their colonizing efforts, that allowed colonizers access to better trade routes and ports of entry in Africa and Asia [26, 27]. The accumulation of such imperial wealth through the usurpation of indigenous property indicates the importance of positionality in trade to nation building and we argue access to nourishment. Further evidenced by the work of Amartya Sen, the great Bengal famines that resulted in the death of over 1.5 million Bengali during British rule was a direct result of empire building inclinations and not a lack of food [28]. The British were able to funnel out of India, food and other resources at the height of World War two that added to their coffers but inflated local food prices leading to mass undernourishment and famine [29]. Trade and nourishment are hence entangled through questions of power, ownership of resources, and political priorities [30].

To further the understanding of global food trade and its impact on national nourishment outcomes, we seek to quantify how positionality in the global grain trade explains why some countries are more nourished than others. Our research asks, how does centrality in the global wheat supply chain affect nourishment levels at the country level? One hypothesis is that counties with higher purchasing power and greater centrality in the global grain network should be better nourished. We focus on global wheat trade given that globally, food grains—wheat, maize, and rice—account for over 50% of human caloric consumption and underpin global food security [5]. Food grains have been important throughout history, having played a critical role in the formation of the earliest states [31] and their expansion as global grain trade routes shaped spheres of political influence. Given the ubiquity of wheat and wheat products in diets globally we use the global wheat supply chain to better understand how positionality in the global wheat trade affects national nourishment outcomes, and how centrality may contribute to or impede undernourishment outcomes. In this research we posit that the more central a country is in the global wheat trade (by trades), the more nourished the populous.

First we model the trade network structure of the global wheat supply chain to measure the centrality or positionality of countries. Harmonization of network characteristics from the social network analysis (SNA) with trade volume, purchasing power, production capacity and geography enables our research to query the relationship between network position and undernourishment outcomes while controlling for geographical influences. We apply the Spatial Auto-Regressive (SAR) model which accounts and adjusts for the effect of neighboring countries on development outcomes. In doing so, we are able to explore not only the impact of trade on the status of undernourishment but also contribute towards a growing understanding of the complexity of the global food system and the need to sustainably build its resilience to shocks. Our study is organized into four sections. First, we describe the research methods, including data collection, and analytical choices while providing context and background for the Social Network Analysis (SNA) Spatial Auto-Regressive (SAR) model. Next, we describe the results comparing with previous studies, followed by the discussion and conclusion sections.

## Research methods and analysis

### Data collection and aggregation

#### 1. Extracting wheat export-import data from the global ComTrade data

We obtained global trade data from annual ComTrade data from CEPII [32]. The 2018 global trade database was analyzed in Stata/IC version 16 software to generate a sub-database of wheat based on 9 wheat codes obtained from the product code data dictionary: 100111, 100119, 100191, 100199, 110100, 110311, 110811, 110900, 230230. These nine codes represent all the wheat related trade as captured in the original dataset (See Table 1). The wheat dataset contains 7, 931 lines of trade flows between 214 countries. The wheat data was further collapsed into two categories—cereals and by-products—to facilitate network analysis. The cereal network comprises of four wheat codes and the by-product network represents the remaining five wheat codes (110100, 110311, 110811, 110900, 230230) found in the global dataset. We find more trade edges for by-products than raw cereals; there are 2,550 cereal edges against 5,381 by-product edges. There are also more countries importing wheat (214) than exporting wheat (146), and only about half the (102) countries grew and exported all the wheat cereals in the global supply chain (See Table 2). We generated a new variable, “region” and assigned each country to one of six regions as per FAO’s designations [8].

**Table 1:** Original wheat product codes and reclassification.

Product	Name	Code number	Recode
Cereals	Cereals: wheat and meslin, durum wheat, seed	100111	1
	Cereals: wheat and meslin, durum wheat, other than seed	100119	
	Cereals: wheat and meslin, other than durum wheat, seed	100191	
	Cereals: wheat and meslin, other than durum wheat, other than seed	100199	
Wheat By-Products	Wheat or meslin flour	110100	2
	Cereal groats and meal: of wheat	110311	
	Starch: wheat	110811	
	Wheat gluten: whether or not dried	110900	
	Bran, sharps and other residues: of wheat, whether or not in the form of pellets, derived from the sifting, milling or other workings thereof	230230	

**Table 2:** Summary descriptive of global wheat supply.

Observations	Number of Trades	Export Country	Import Country
Total	7931	146	214
Wheat Cereals	2550	102	178
Wheat By-Products	5381	141	214

#### 2. Creating node and edge tables for SNA

Networks consist of “nodes”, denoting the actors in the network, and “edges” are the relations linking the nodes. Nodes include all countries involved in the export and import of wheat and wheat products. The node table was extracted from the wheat sub-database we created and contained unique country id, name, latitude and longitude. For each country, latitude and longitude were generated using Google maps geo-locator, with two exceptions. CEPII aggregated trade data for the South African Customs Union (SACU) countries: South Africa, Namibia, Lesotho, Botswana and Swaziland. It is represented in the data set as ID 711. ID 490, listed as “other Asia”, makes reference to Taiwan’s trade data and we use Taiwan’s geographical information for the SNA. We include the SACU trade data and use South Africa’s geographical data (longitude and latitude) for the social network analysis (SNA). The edges in the network were represented by the 7, 931 lines of trade flows. Each line of trade flow included information on exporting and importing country ID’s, volume and value of wheat export and import by respective countries, and the product code. Product codes were assigned to each country based on original wheat code (See Table 3) using Stata script. We included all edges and nodes that represented wheat trade flows. Separate node and edge tables were created for SNA.

**Table 3:** Summary descriptive of global wheat supply.

	Number of Trades	Countries Trading
	Edges	Nodes
Wheat—Total	7931	215
Wheat Cereals	2550	178
Wheat By-Products	5381	214

#### 3. Extracting development indicators from the world development indicators database

The world development indicators [33] database is longitudinal in nature, capturing 66 indicators spanning 60 years (1960–2020). Our dataset contains data for 213 countries, and 17 development variables for 2018. Given we are interested in understanding how food production capacity affects undernourishment we generated a new variable, “agricultural productivity”, that is a ratio of agricultural land and total population There were a few exceptions. For countries that did not have agricultural land data available for 2018, we used the last data point available (2016). In the case of Eritrea, we used 2011 population data from the WDI database, as population data is not available 2011 onwards.

#### 4. Merging trade and development data

Next, prior to merging trade and development data, we aggregated the 7, 931 lines of trade flows by country, by summing total quantity and value of wheat exports and imports. We also created 4 variables to capture desegregated wheat data for raw cereals and by-product export and imports. The process yielded a dataset of 201 countries: 14 countries, mainly island nations (Christmas Island, Cook Islands, Falkland Islands, Other Asia (Taiwan), Bonaire, Niue, Norfolk Islands, Saint Barthélemy, Saint Helena, Anguilla, Saint Pierre and Miquelon, Southern African Customs Union, Tokelau, Wallis and Futuna) were dropped from the trade dataset as they did not have development data recorded for their respective countries, and 12 countries from the development dataset were dropped as there was no trade data recorded for these countries in our database: Botswana, South Africa, Swaziland, Lesotho, Namibia (South African Customs Union Countries), Lichentenstein, Luxembourg Puerto Rico, Virgin Islands, and Faroe Islands. For the final analysis, our dataset contains 141 countries, owing to 55 countries missing values for the outcome variable–undernourishment and five countries missing values for purchasing power parity.

### Analytical methods

#### 1. Social network analysis

The SNA software package Gephi was used to visualize the network graph and run descriptive analysis for an undirected network [34]. We used the undirected network to avoid giving preference to the targets (importers) and to better measure trade relationships as bi-directional as opposed to unidirectional (from exporter to importer). Due to the nature and structure of trade links–more countries import and only half the countries in the dataset export wheat cereals and by-products–we opted for an undirected network. A directed network could potentially create bias in the centrality measures given the unbalanced nature (more importers than exporters) of the global wheat trade. Additionally, an unweighted network structure was selected to assess the importance of the number of trade links. The network was visualized using the Fruchterman Reingold projection, which places nodes connected by an edge in relatively close proximity with one another [35].

Hubs in social networks can be measured in many ways. The general principle is that if ‘all paths lead to Rome’, Rome is an important hub that can influence the rest of the system. We calculated, eigenvector and betweenness centrality, and total degree as measurements denoting country’s influence in the global wheat supply chain. Eigenvector centrality measures how well connected each node (Country) is to other influential nodes (countries), computing power and status of respective countries and their connections [36]. Eigenvector centrality can be useful in identifying important secondary markets. Countries that do not have enough land to produce food to support their populations often act as important manufacturing locations, importing raw products and wooing processors with low-cost labor to produce processed byproducts. These secondary market hubs need to be well-connected to other countries of influence to maintain their status. The more well connected a country, the greater the extent of its centrality and power of influence [37]. It follows that the centrality of a given node *i* is equivalent to the sum of the centralities of its neighbors [36]:

Equation 1:

$$\sigma_{i}=\frac{1}{\lambda }\sum_{j=1}^{n}a_{ij}\;\sigma_{j}$$

where *σ*_*i*_/*σ*_*j*_ represent the centrality of nodes *i/j*, and λ is the largest eigenvalue of the adjacency matrix *a*_*ij*_, and *a*_*ij*_ is 1 if nodes *i* and *j* are connected and 0 otherwise. However, in an undirected network with no loops, the main diagonal is 0 [37].

On the other hand, betweenness centrality measures the percentage of shortest paths that pass through a node [36]. Countries with high levels of betweenness occupy positions of importance in the global wheat supply chain, as they are points of critical connection for exporters and importers, connecting different regions or clusters within the network. Betweenness centrality of a vertex υ can be measures as followed [37]:

Equation 2:

$$\upsilon =\sum_{s\neq \upsilon \neq t\in V}\frac{\delta_{st}(\upsilon )}{\delta_{st}}$$

where *δ*_st_ is the total number of shortest paths from node *s* to node *t*, and *δ*_st_(υ) is the number of shortest paths passing through vertex υ [37].

Degree measures the total number of trade relationships each node (country) has in the global wheat supply network, and higher the degree, the more central the country in the distribution of wheat. Degree (k_*i*_) is computed formulaically as Equation 3:

$$k_{i}=\sum_{j\in \Pi (i)}a_{ij}$$

where *a*_*ij*_ is the adjacency matrix for nodes *i* and *j*, and *Π(i)* is the neighborhood of node *I* [37].

Alternatively, many countries have taken a self-sufficiency approach to hunger and food security since the 1970s, enacting policies to ensure they preserve farmland and can produce enough food to feed their population. Countries with low degree could potentially fit this description. Such policies may provide resilience from economic shocks, but may similarly leave the country vulnerable to natural disaster shocks where domestic food production capacity may be wiped out without alternative food supply.

#### 2. Spatial Autoregressive Regression (SAR) analysis

We use a cross-sectional model to study the effect of trade, purchasing power, population and land dynamics on undernourishment globally, while controlling for regional variation, in 141 countries that bought and sold wheat grain and wheat products in 2018. We first test the presence of spatial correlation of errors in the dataset. Spatial proximity can affect the values of measurements in nearby areas; indeed, closer the geographical areas the more correlated their measurements might be [38]. Given that our data contains spatial elements, independence of observations could not be assumed, prompting the test for spatial correlation of errors using STATA’s inbuilt Moran’s test for spatial dependence [39]. Following a confirmation of spatial correlation, we fit a spatial autoregressive model to estimate the impact of centrality and trade (network characteristics of global wheat supply chain and quantity of wheat exported and imported), population and land dynamics (region, agricultural land per capita), and purchasing power (gross national income per capita) on hunger (percentage of population undernourished).

Model diagnostics, which explore the structure and skewness of data, outliers, robustness of errors, risk of multicollinearity and heteroskedasticity showed that generalized method-of-moments estimator also known as the spatial two-stage least squares (gs2sls) was best fit for the model. Model diagnostics also revealed a high degree of multicollinearity when multiple measures of network statistics were included in a single model. We included two network metrics, eigenvector and betweenness centrality, each in a separate model see (equation 1 and 2) to see how different measures of centrality affect undernourishment. Additionally, based on the literature we chose a combined inverse distance continuity weighted matrix for the analysis [39]. A combined inverse distance contiguity matrix, is a weighting matrix that contains inverse distance for neighbors and 0 otherwise, created by multiplying individual inverse distance and contiguity matrices element by element [39]. Given that parts of the world are closely connected (contiguous continental countries that share a border), while others are not (islands), a matrix that accounts for close proximity and inverse distance was selected.

In the SAR regression, we regressed undernourishment, trade, population and land dynamics, and purchasing power covariates to fit the equation below:

Equation 1 (spatial lag on error term with interaction term): y = β0 + β1x1 + β2x2+ β3x3 + (I − ρW) ^−1^ ɛ

Equation 2 (spatial lag on error term): y = β0 + β1x1 + β2x2+ (I − ρW) ^−1^ ɛ where y is the undernourishment coefficient (λ), β0 is the intercept, x1 is a matrix of observations on country level centrality, wheat imports and exports, and purchasing power covariates, x2 is a matrix of country control variables (region and population and land dynamics), x3 is the interaction term (the effect of eigen centrality on undernourishment, given different levels of purchasing power), ϵ is random error, and β1, β2, and β3 are vectors of parameters to be estimated. W is the spatial weighting matrix (combined inverse distance continuity) and ρ is the spatial autoregressive coefficient. Dependent variable, undernourishment is log transformed.

### Summary statistics

The dependent variable, undernourishment, indicates that on average 9 percent of the population across the countries suffer from hunger. Explanatory variables used in the study comprise centrality, trade, purchasing power, land and population dynamics. Wheat supply chain network characteristics are derived through social network analysis and include eigenvector and betweenness centrality measures. Eigenvector centrality measures capture not only the number but also the importance of each country’s trading partners, while betweenness centrality measures individual importance based on the number of shortest routes assigned to the node. Trade variables include the quantity of cereal grains and wheat by products exported and imported in metric ton. Gross national income per capita accounts for national purchasing power parity against the international dollar and production capacity is a ratio of agricultural land (square km) per capita for each respective country. We also include a categorical variable that denotes 6 different regions of the world to control for location and geographical similarities and influences. Among the 146 countries we find that countries are moderately connected (eigenvector of 0.35 and betweenness centrality of 161.79 on average) to powerful wheat exporters and that there is substantial variation in the trade network. The average gross national income per capita is $20,809 and countries have about 0.012 square kilometers of agricultural land per person for food production on average. On average, countries exported 1.3 million metric tons of wheat cereals and 159,233 metric tons of wheat byproducts, and imported 1.2 million metric tons of wheat cereals and 145,746 metric tons of wheat byproducts in 2018. See Table 4 for more details.

**Table 4:** Descriptive statistics.

*Category Name*	*Variable*	*Short Variable Name*	*Units*	*Descriptive Statistics*	*n*	*Data Source*
*Dependent Variables*						
*Hunger*	Undernourishment	UN	Percentage	8.89 ± 9.27	141	World Bank World Development Indicators; 2018
*Explanatory Variables*						
*Wheat Trade Network Characteristics*	Eigencentrality	EC		0.35 ± 0.25	141	BACI: International Trade Database at the Product-Level; 2018
	Betweenness Centrality	BC		161.79 ± 415.25		
*Wheat Exports*	Cereal Grain Export	CGE	Metric Ton	1328724 ± 5154672		
	Wheat By-Product Export	BPE		159233.8 ± 429579.4		
*Wheat Imports*	Cereal Grain Import	CGI		1250308 ± 2125639		
	Wheat By-Product Import	BPI		145746 ± 318947.6		
*Purchasing Power Parity*	Gross National Income per capita	GNIPC	Current International $	20809 ± 18695		World Bank World Development Indicators; 2018
*Population and Land Dynamics*	Production Capacity per capita	PC	Square km of ag land per person	0.012 ± 0.035		
	Region	RG	Categorical Data	2.97 ± 1.64		Food and Agriculture Organization, 2020
			Africa = 1			
			Asia = 2			
			Latin America & Caribbean = 3			
			North America = 4			
			Europe = 5			
			Oceania = 6			

## Results

### Social network analysis of the global grain trade

**Fig 1:**
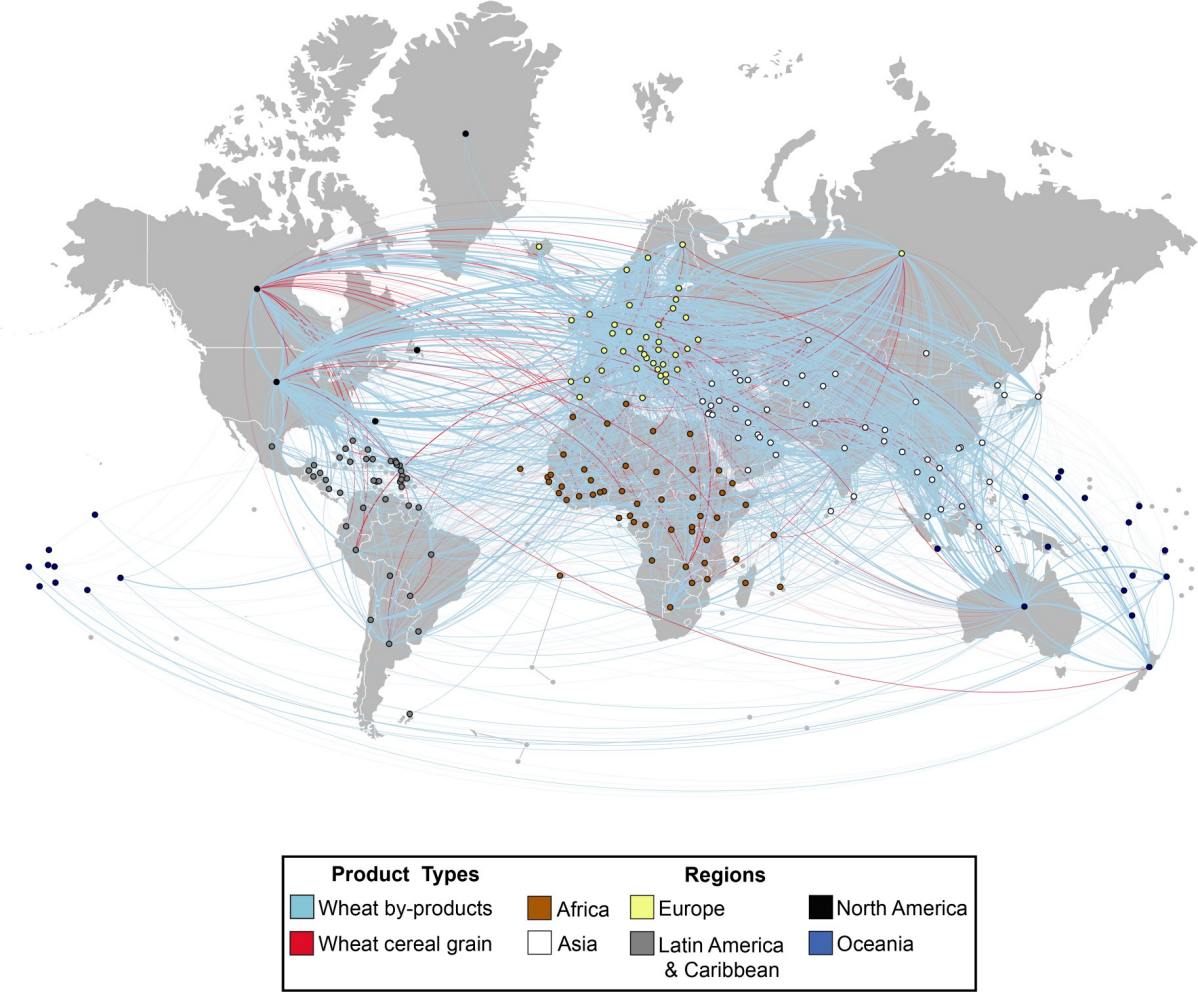
Social network visualization of global grain trade.

Networks consist of “nodes”, denoting the countries in the network, and “edges” are the trade relations linking the nodes. The global grain trade network we use consists of 215 countries with 7931 trade relationships for wheat and wheat byproducts based on 2018 ComTrade data. About 86% of the trade networks involve byproduct (blue), only 14% is based on raw products (red) (See Fig 1).

The undirected network architecture indicates that a few countries control the global wheat supply. In fact, the six most central countries (by betweenness centrality and eigenvector centrality) account for more than half of all wheat (in metric tons) exports globally: Germany, Italy, France, Turkey, Russia, the United States, and Canada when considering betweeness centrality. Countries that are central to the global wheat supply chain are not necessarily the largest exporters of wheat and wheat by-products: Italy ranks 24^th^ by total export quantity and Turkey ranks 12^th^ by the same measure for example. Conversely, Australia and Argentina are ranked fifth and sixth by export volume, but have much lower centrality. Additionally, most central countries (by trades, not volume or value) are in North America and Europe, with the exception of Turkey, India, and Australia. The concentrated centrality of a few countries in global wheat trade by number of trades (and volume/value) demonstrate that disruption in only a few countries would have broad impacts. Indeed, this makes the global wheat value chain very vulnerable as a shock to one of these countries is likely to propagate across the globe.

**Fig 2:**
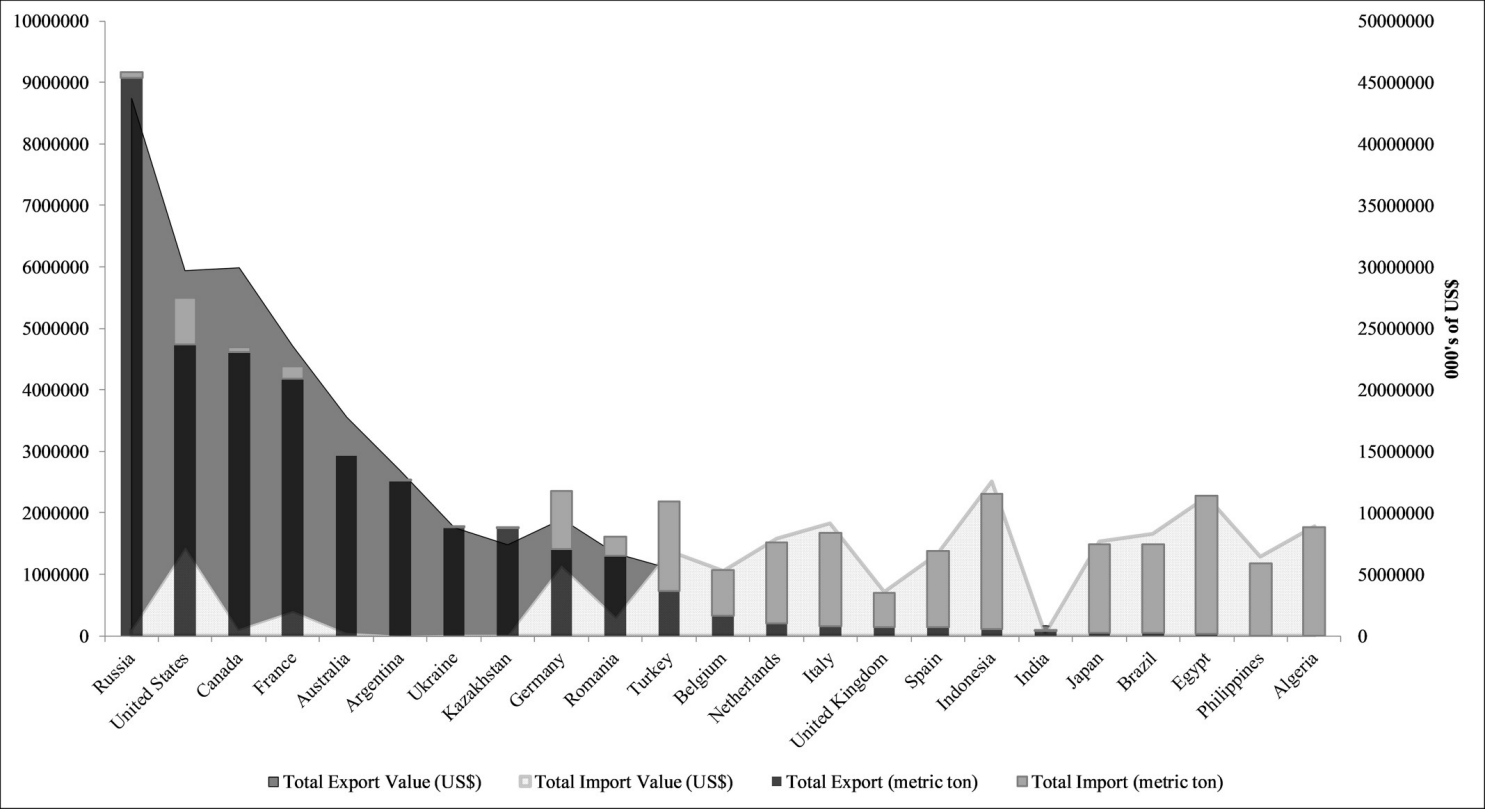
Top 10 countries by volume and value of trade.

**Fig 3:**
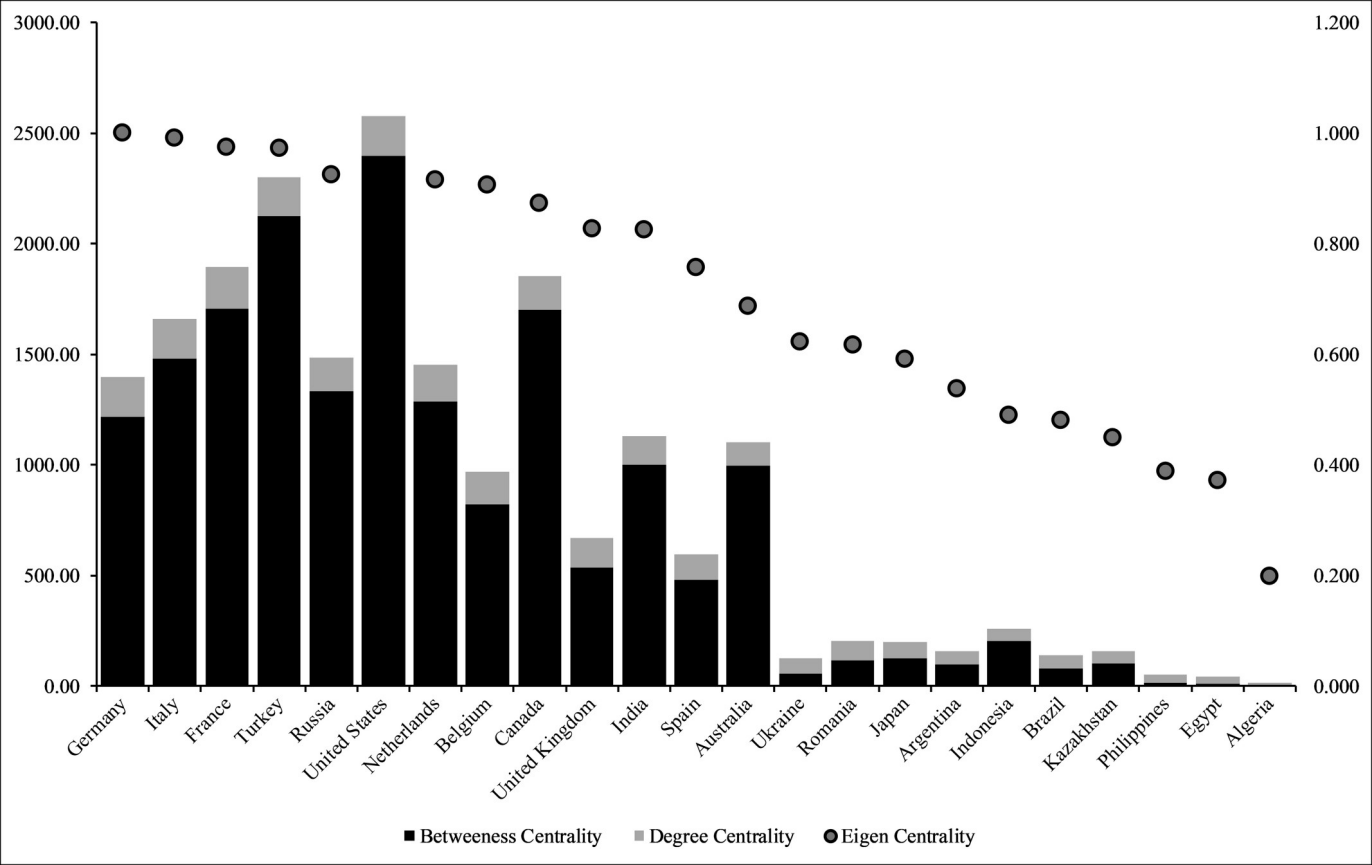
Top 10 countries by centrality measures.

If we were to assess the importance of countries in the global wheat trade by volume and value of trade, there would be a significant difference in results. Figs 2 and 3 below illustrate differences in country importance based on centrality measures versus volume and value. When using volume and value, Russia is the top exporter in the global wheat trade, followed by Canada and the United States. In comparison, centrality measures point to the importance of Germany, France and the United States. Our analysis also shows that while Australia and Argentina are important wheat exporters, they are not as critical by centrality measures. Even when we assess the global wheat trade by value and volume, European and North American countries retain their control over the supply chain. Though the list of most important European countries change when we compare centrality results versus volume and value: Kazakhstan, Romania, Ukraine (volume and value) replace Belgium, Netherlands, and the United Kingdom (centrality) among the top ten most important countries. On the import side, Egypt, Indonesia, and Algeria are top importers, but feature low with respect to the centrality measures. While Asian and African countries hold top spots for wheat imports, there is more diversity in the regions that control the demand side of the wheat supply chain. Of interest in the global wheat trade are Italy, Turkey, and the Netherlands, who make the list of top ten importers and all three centrality measures. In other words, in addition their high trading power (volume and value), these countries have also the potential to influence the whole global wheat supply chain because of their influence extends beyond their direct trading partners.

The centrality measures point to the importance of countries who are critical to the supply chain, beyond quantity and volume. Centrality measures point to the importance of and the effect of early nation states establishing supply routes and also the later empire building efforts of these states—Belgium, Germany, Italy, Netherlands, and the United Kingdom—through colonization [27]. With the exception of Germany, France and Russia, the other top European countries (based on volume) by centrality measure are not significant exporters of wheat and wheat by-products. Their importance in the global wheat chain is however elevated due to their roles as conduits of trade: having powerful trading partners (eigenvector), and being along the shortest route (betweenness) for trade to occur, which all contribute the dynamics of global wheat trade. Like in Europe, some Asian countries such as India and Turkey occupy central and critical positions in the wheat supply chain even though neither is a top exporter or importer of wheat by volume. Turkey though ranks ninth, behind the United States in import value of wheat and wheat by-products. The Ottoman empire has a long history of building trade routes and India through the British occupation, and beyond cemented their place as an integral trade route owing to their geographical location and supply of cheap labor to lower costs of supply-chain logistics. Turkey’s proximity to the Black Sea, giving it access to low-cost milling quality wheat, has helped the country develop a pivotal role in the region’s market [40].

**Fig 4:**
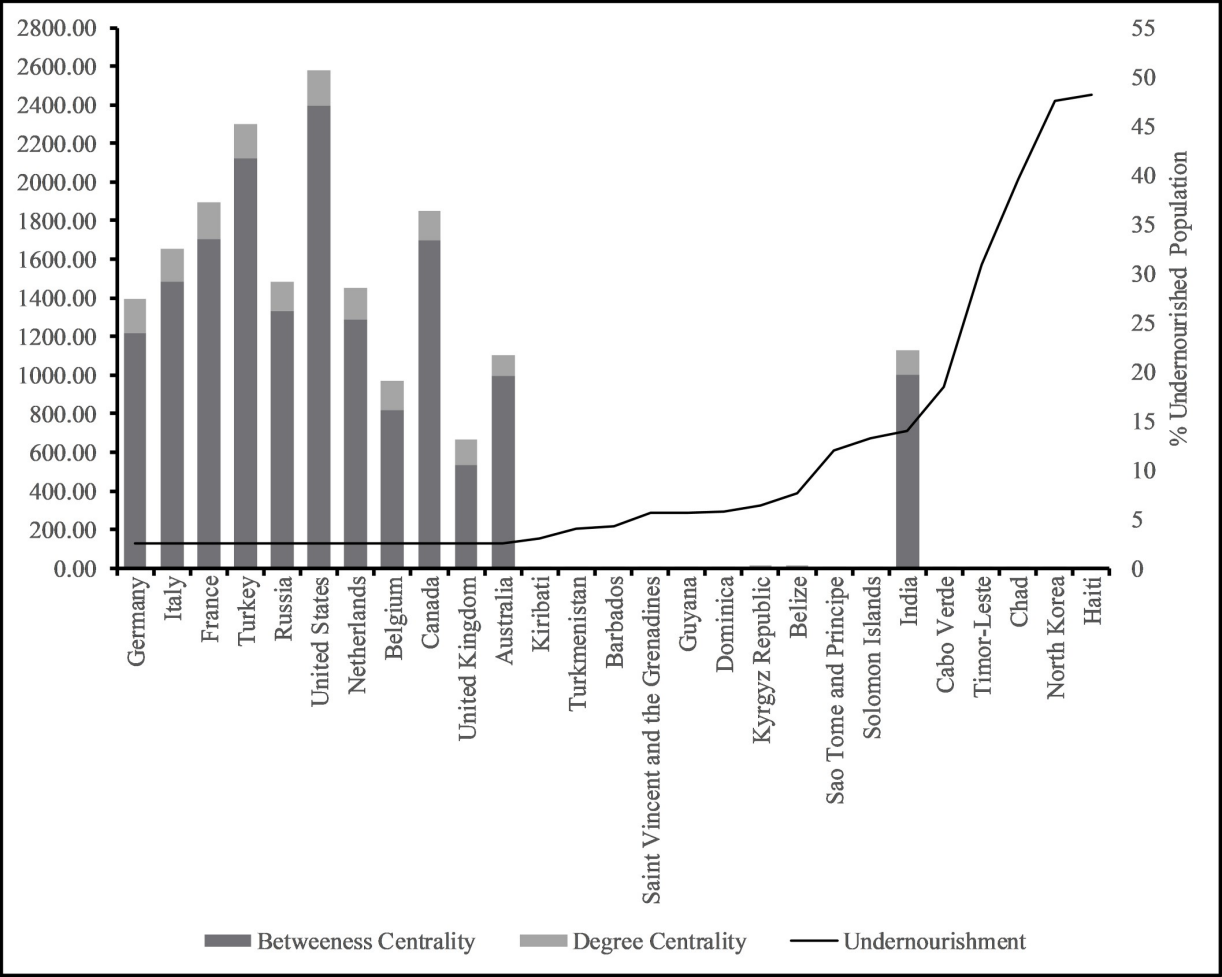
Top and bottom 10 countries by centrality measures.

**Fig 5:**
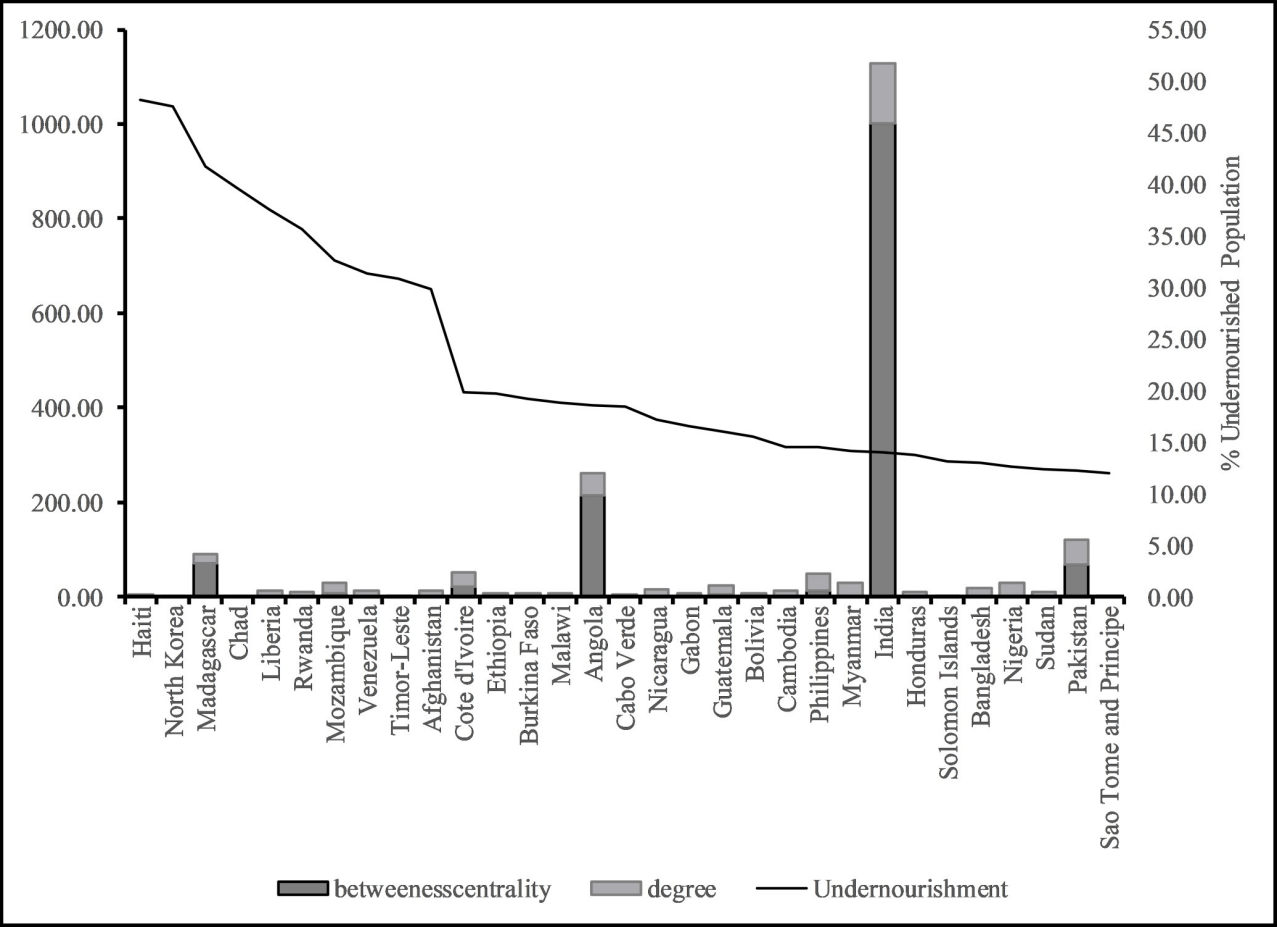
Very highly and highly undernourished countries and their centrality measures.

In this paper we posit that the more central a country is in the global wheat trade the more nourished the populous. For example, China and India each consume 17–18% of global wheat [41] and on average, 500 kcal of food energy per capita per day comes from wheat [42]. We find that countries with very high (15 countries) and high levels of centrality (21 countries), also have very low to low levels of undernourishment, with the exception of India (See Fig 4). In contrast, countries with very high to high levels of undernourishment (36 countries), have low and very low levels of centrality in the network (total degree and betweeness)—with the exception of India, again (See Fig 5). Low, average, high, and very high levels were created using one standard deviation below mean, at mean, one standard deviation above mean, and two standard deviations above mean respectively. The global wheat trade network also highlights outliers. For example, Samoa and Montenegro experience very high levels of nourishment but are not central to the global wheat network. Montenegro is considered a high-middle income country and ranks high (48) on the Human Development Index [43], indicating the country’s wealth and purchasing power may enable it to maintain low levels of hunger, despite its low centrality. Samoa on the other hand, is an Island nation in the South Pacific where subsistence agriculture and fishing contribute to nourishment (a third of all food consumed) which might explain why its low centrality in the network is not negatively correlated to national hunger outcomes [44]. Overall, countries in the Global North are central to the network and are nourished, while countries in the Global South are less central to the global wheat network and experience higher levels of undernourishment. Similar results for nourishment and location were obtained by colleagues in their evaluation of global food security [45].

Following the FAO, in this paper, we use undernourishment as the main hunger indicator that measures the share of the population whose caloric intake is insufficient to meet the minimum energy requirements necessary for a given population [8]. Whereas food insecurity is when people lack physical and financial access to adequate amounts of safe, nutritious and culturally appropriate foods, necessary for development and an active life [8]. Severe food insecurity is strongly related to undernourishment and about 9% of the global population is undernourished and experience severe food insecurity [46]. We posit that for our analysis, undernourishment is a good measure of how food secure a population is based on nutrient intake. According to the 2021 Global Hunger Index [47], most countries that will not achieve a low level of hunger by 2030 are located in Africa South of the Sahara, with the remaining countries spread between South Asia, West Asia and North Africa, East and Southeast Asia, and Latin America and the Caribbean.

When combining food production potential, agricultural land availability and centrality measures, we find, countries in the Global North like Belgium and Netherlands, with very little agricultural land per capita are still highly nourished, and have very high centrality measures in the wheat network (see Fig 4). As discussed earlier, Belgium and Netherlands established their centrality and control of the supply chain through the colonization of Global South Countries [28]. While Belgium’s interests mainly targeted African countries (Congo DRC, Rwanda, Burundi, and Tangiers) [48], the Dutch were far more prolific in their efforts and established colonies beyond Africa, in Asia, North America, and Europe as well [49]. These trading routes long established on the backs of the slave trade and colonial rule, allow countries such as Belgium and Netherlands to be nourished through their exertion of path dependent influence, despite their own lack of production capacity [26, 27]. Other countries that exhibit similar levels of nourishment despite production capacity, also tend to have high degrees of centrality in the wheat network—with Samoa being the exception. These Global North countries are able to buy into the wheat supply chain given their positionality adjacent to important wheat export hubs.

**Fig 6:**
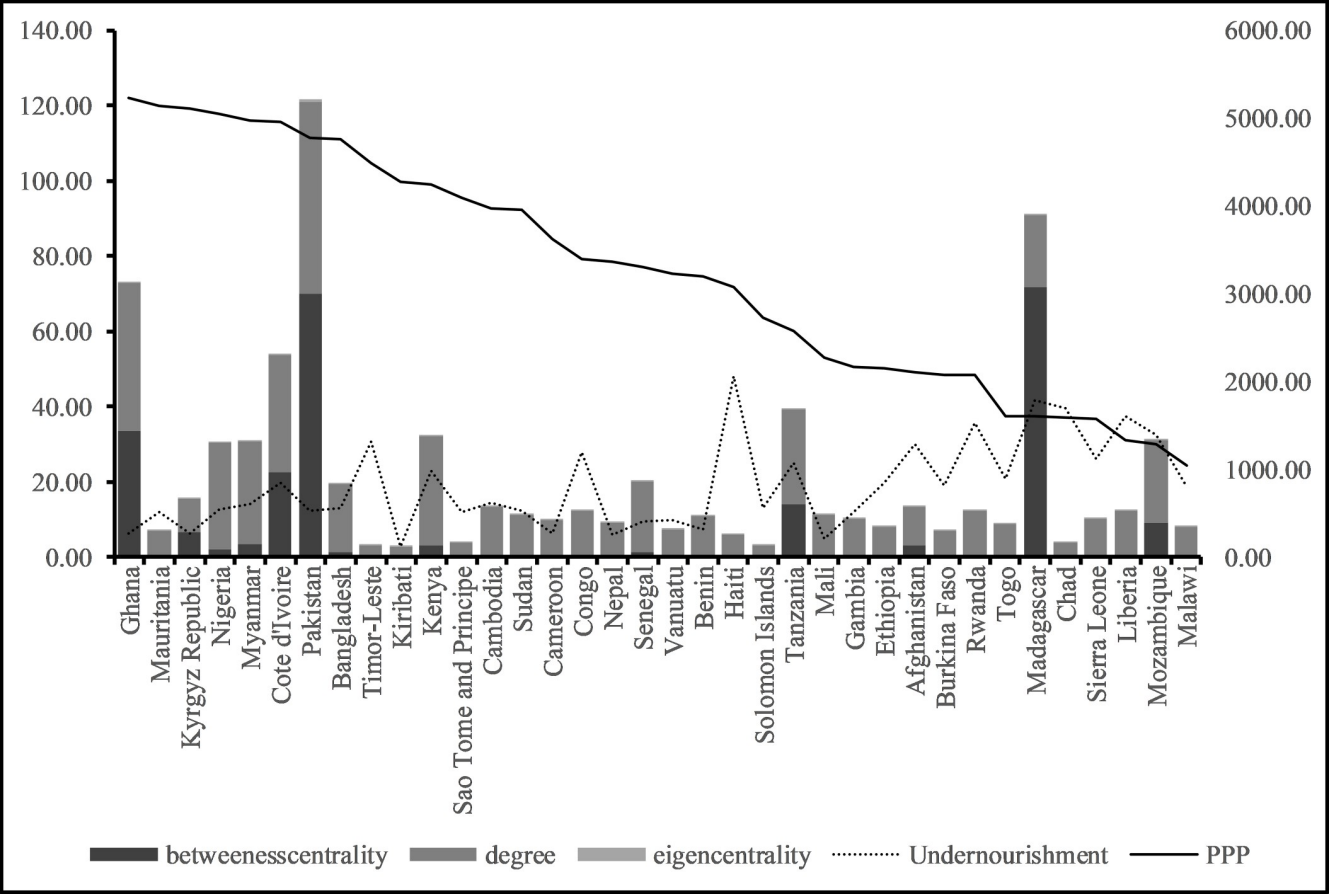
Countries with low purchasing power, undernourishment and their centrality measures.

Purchasing power also plays a role in who has access to the global wheat markets. We find that among countries with low purchasing power, none have a high or very high degree of centrality. In general, countries with low purchasing power are located in the Global South and often suffer from high and very high levels of undernourishment—with the exception of a handful of countries in West Africa: Gambia, Ghana, Mali, and Senegal (See Fig 6). The relationship between purchasing power, undernourishment, and centrality does not appear to be linear in countries with low purchasing power and low centrality.

### How does the global wheat trade affect national food security?

We use spatial analysis to understand the complex relationship between levels of undernourishment and economic and trade characteristics influenced by geography and positionality in the wheat trade network. We also include a second model to assess the impact of multiple measures of centrality in the global wheat network on undernourishment (see Table 5).

**Table 5:** The effect of trade, purchasing power, land, and wheat imports on undernourishment.

	Model 1			Model 2		
	Estimate	SE	p-value	Estimate	SE	p-value
GNIPC	-0.000035*	5.49e-06	p<0.001	-0.00002*	2.93e-06	p<0.001
CGE	-0.000067	0.000059	0.259	-0.0001*	0.00005	0.017
BPE	-0.0025*	0.0007	0.001	-0.003*	0.0007	p<0.001
CGI	-0.0006*	0.0003	0.024	-0.0007*	0.0002	0.005
BPI	0.003*	0.001	0.037	0.004*	0.001	0.013
PC	2.06*	0.67	0.002	2.32*	0.62	p<0.001
RG						
1	0.79*	0.19	p<0.001	0.81*	0.18	p<0.001
2	0.48*	0.12	p<0.001	0.41*	0.12	0.001
3	0.34*	0.16	0.032	0.31*	0.14	0.032
4	-0.03	0.14	0.852	0.07	0.19	0.711
6	-0.23	0.21	0.288	-0.22	0.21	0.298
EC	-0.79	0.51	0.121			
BC				0.0003*	0.0001	0.048
EC x GNIPC	0.000035*	0.00001	0.001			
Constant	2.19*	0.22	p<0.001	1.98*	0.14	p<0.001
**ρ**	0.58*	0.23	0.012	0.68*	0.20	0.001
Pseudo R-Squared	64.41%			62.24%		

* indicates statistical significance at p = 0.05.

First, where people live matters. Our findings indicate that relative to countries in Europe, undernourishment is more pronounced in Asia, Africa, and Latin America and the Caribbean. Undernourishment is expected to be 2.2, 1.6, and 1.41 times higher, if living in a country in Asia, Africa, or Latin America and the Caribbean, respectively, relative to living in Europe. Undernourishment does not significantly differ for countries in North America, and Oceania, relative to countries in Europe.

Second, whether countries exported or imported raw wheat or wheat byproducts also affect their undernourishment. We find for every additional 10,000 metric ton of cereal grain that countries import, there is, on average, a significant decrease of 0.06 percent in undernourishment while every additional 10,000 metric tons of wheat byproducts that countries import is associated with a 0.31 percent increase in undernourishment. As an explanation, grains are cheaper per caloric value compared to processed by-products such as flour, breads, and other value added products. Thus, cheaper grains are more affordable than value added wheat byproducts, and more accessible to people of low economic status relative to value added wheat products. Our results consistent with others [25], find that lower income countries are not meeting their nutrient needs through trade. Instead of nutrient dense products, cereal grains dominate imports which do not significantly improve nutritional adequacy. Additionally, when countries exported 10,000 metric tons of wheat by products, there was an associated 0.25 percent decrease in undernourishment rates. The model does not show a significant relationship between cereal grain exports and undernourishment rates. These results validate the SNA results. The SNA shows Europe as the center of cereal grain exports, and these countries are seemingly nourished relative to those in Asia, Africa, and Latin America and the Caribbean, and thereby increasing cereal grain exports is unlikely to affect undernourishment. However, countries with higher rates of undernourishment also happen to have lower purchasing power, and lower labor costs, who are then able to capitalize on the secondary grain markets through value-adding and manufacturing of flours, breads and other related products. Thereby the export of wheat byproducts is related to decreased rates of undernourishment.

Third, the amount of agricultural land per capita (production capacity) appears to be only marginally significant in the moderated spatial regression and is inversely related to undernourishment. Undernourishment is expected to be 7.85 times greater for every one-unit increase in agricultural land per capita. By way of explanation, the availability of land is just one factor of production. Who owns the land and the related productivity of the lands is another matter. Given the global nature of land ownership, and recent examples of land grabs in the African continent and elsewhere by foreign investors, increase in the availability of agricultural land may not equate with food production for the nation. For example, in 2014, the island nation of Kiribati, purchased a 5,500-acre plot of arable land on Fiji’s second largest island with the intention of developing the land for agriculture for people in Kiribati [50]. The land purchase, once fully developed for farming, will displace food production for 500 Fijians who currently live on and use the land to farm [51]. Regardless of the internal displacement issues surrounding the land purchase, the food produced will not generate nourishment or economic benefits for Fiji, but Kiribati instead.

**Fig 7:**
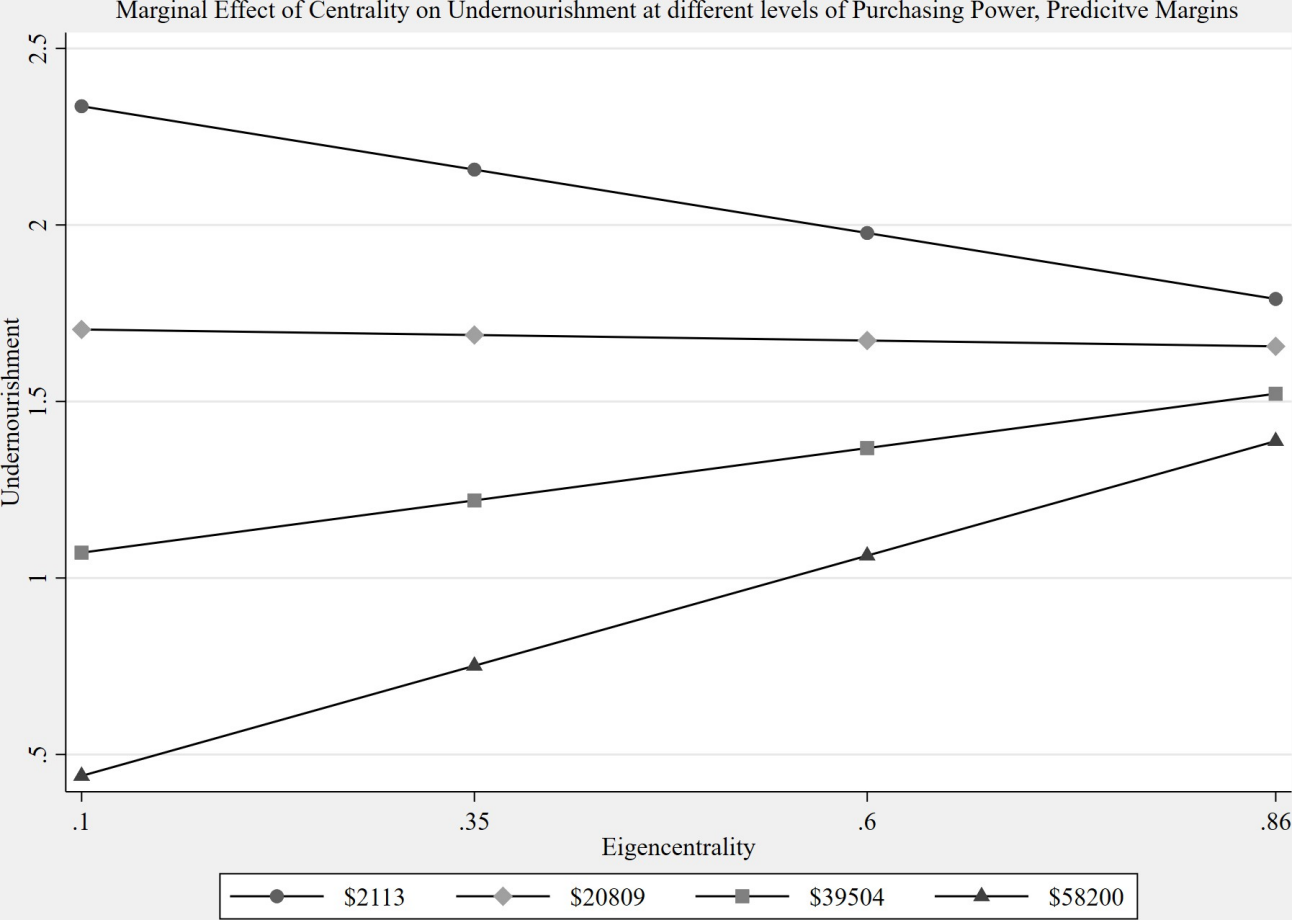
Predicted values.

Fourth, centrality matters but only when considered relationally to purchasing power in this model. In model 1, the two-way interaction (purchasing power x eigen centrality) is statistically significant. The interaction coefficient indicates the moderated effect on undernourishment is positive. The positive relationship suggests that eigen centrality moderates the effect of purchasing power in an extractive manner. Given the positive interaction coefficient we expect that as eigen centrality and purchasing power among countries increase, that their undernourishment rates will increase as well, which is counter-intuitive to our hypothesis. To this end, we estimated predictive margins for the interaction, depicted below (Fig 7), to assess the simple effect of eigen centrality on undernourishment for countries that had low ($2,113), medium ($20,809), high ($39,504), and very high ($58,200) levels of purchasing power. Low, average, high, and very high levels were created using one standard deviation below mean, at mean, one standard deviation above mean, and two standard deviations above mean respectively.

Among countries with low and medium levels of purchasing power, eigen centrality did not have a statistically significant effect on undernourishment. For countries with high levels of purchasing power, eigen centrality was positively associated with undernourishment rates, but this effect was only marginally significant. However, at very high levels of purchasing power, we found eigen centrality to be positively associated with levels of undernourishment. For countries with high and very high levels of purchasing power parity, for each unit increase in eigen centrality, undernourishment is expected to be 1.8 and 3.48 times greater respectively. Our results, while diverging from our hypothesis, are similar to others who found that super exporters and importers erode the resiliency of the food system [13]. High levels of purchasing power parity allows countries to easily take advantage of their centrality in the food supply chain and engage in high levels of trade, that then behaves extractively, and undermines national nourishment.

Next we looked at the simple effect of purchasing power parity on undernourishment for countries that had low (0.1), medium (0.3), high (0.6), and very high (0.86) levels of eigen centrality. Among countries with low levels of eigen centrality, purchasing power parity was negatively associated with undernourishment. Similarly, among countries with average and high levels of eigen centrality purchasing power parity was negatively associated with undernourishment, respectively. At very high levels of eigen centrality, purchasing power parity did not have a statistically significant effect on undernourishment rates. We see that for countries with low, average and high levels of eigen centrality, for each unit increase in purchasing power parity, undernourishment is expected to be 0.003%, 0.003% and 0.002% lesser respectively. These results are consistent with the simple effect of eigen centrality on undernourishment.

In model 2, we find that betweenness centrality is positively associated with rates of undernourishment. When we consider the shortest route, as the centrality feature in the wheat network, with all else constant, a one-unit increase in betweenness centrality increased undernourishment in countries by 0.03%. The increase in shortest route would enable easier access to trade markets, allowing countries to engage in more trade for wheat products, more easily. Overall, increased trade integration is expected to stabilize food prices, boost returns to farmers and reduce the prices faced by consumers [52]. However, when these transmission mechanisms fail, increased access to food trade may lead to increased undernourishment as shown in our results. Trade is not magic bullet to guarantee food security by itself; it should be part of a comprehensive and coherent policy package to achieve food security [53].

We also find that for every 10,000 metric ton of cereal grain that countries export, there is a 0.01 percent decrease in undernourishment on average. In all other aspects model 2 is similar to model 1.

## Discussion

Our work sheds light on how trade, and centrality in the global wheat trade network affects national nourishment food security outcomes. Consistent with other trade network studies [10, 12] we find that very few countries exert high levels of centrality over the global wheat trade supply chains. Importantly, we find that the centrality of these countries, as opposed to volume of wheat produced or traded, determines their influence in the wheat supply chain. European and North American countries have the highest centrality in the global wheat trade network, and therefore significant power by virtue of their positionality. We also find that countries in the Global North who are central to the network, are less likely to suffer from malnourishment, while countries in the Global South are less central to the global wheat network and experience higher levels of undernourishment. These results align with other studies [1, 11, 16, 20, 21] that found low resourced, and developing countries are more adversely affected by shocks when they happen. Conversely, Global North countries with low production capacities are able to control a significant share of the wheat supply chain given their purchasing power and location adjacent to important wheat trade hubs. These countries include Belgium, Netherlands, Italy, and the United Kingdom. We argue that the supply chain structure that favors the Global North represents a high risk of food insecurity to the Global South countries. The current inflation in global food prices tied to the war in Eastern Europe affecting exports from two critical wheat growing countries is a harbinger of the vulnerabilities in the global food system with centralities concentrated in a handful of countries. A more distributed network structure [10], could be achieved by diversifying trading partners away from these central actors; thereby creating a global grain supply that is less vulnerable to shocks.

The parametric component of our analysis confirms that trade, and centrality in the trade network have significant implications for national levels of nourishment. While previous studies have suggested that the globalization of the food system through increased trade flows is harmful to national food security [54, 55], our findings suggest that for countries with low purchasing power, trade allows improvements in nourishment levels but for countries with very high purchasing power, trade can increase hunger outcomes. These findings are consistent with others that illustrate super-exporters and importers erode resiliency in the global food system and may even erode their own resilience, contributing to higher levels of undernourishment [13]. Indeed, for higher income countries trade is extractive in nature, with agricultural products leaving local communities for more lucrative international markets. For countries that are super-importers the reliance on trade to fulfil national nourishment outcomes may fall short, as others have found [25]. Our results suggest that extensive exports may contribute to increased delocalization and destabilization of national food systems, contributing to overall decrease in modularity [7, 13, 14].

Depending on the measure of centrality, the results suggest that the effect on undernourishment varies. While eigenvector centrality was important in relation to purchasing power parity and had both a hunger reducing and increasing effect, betweenness centrality adversely affected undernourishment rates. The differences in impact on undernourishment rates illustrates the importance of understanding the many ways centrality can be measured, what it measures. In our case, there is a differential effect between eigenvector centrality (captures critical secondary processing hubs like India and Turkey), and betweenness centrality (captures important transit hubs like the Suez Canal in Egypt).

We also found that the more agricultural land does not necessarily translate to higher levels of national nourishment. Globalization has not just outsourced food supply chains but also the means of production. Fluidity in national regulations and market systems have allowed vast swaths of land to be bought and traded by actors foreign to local communities. As a result, powerful entities are able to extract resources from faraway places without having to build large supply networks. The food resources are simply plugged into the vast globalized food chain with little to no positive impact on communities where the chain started. Who owns the means of production, rather than where the factors of production are located seems to be the critical factor. Recent trends indicate the distribution of hunger in the world will change substantially, making Africa the region with the highest number of undernourished in 2030. Indeed, despite longstanding recognition of the benefits of trade and its importance of improving food security, Africa is still performing beneath its potential in global and regional agricultural markets [56, 57].

To correct the unbalanced structure of the trade network as highlighted in this paper, our findings call for more emphasis on regional and localized food systems because well-functioning local food systems are able to counter shortfalls and perturbations in the larger globalized food system. In addition to the benefits of global trade, intra-regional trade has been increasingly recognized as a key element of efforts to increase food security [57]; in Africa, leaders committed to tripling intra-African trade in agricultural commodities and services by 2025. This includes the establishment of a continental free trade area and a continental common external tariff with measures to increase investments in trade infrastructure [56].

## Conclusion

Global food trade accounts for a quarter of all food produced for human consumption is traded globally [5]. Food trade has become an essential component of meeting caloric and nutritional adequacy but has also opened economies up to shocks and extraction of food resources. With this research, we assess how arable land per capita, purchasing power parity, food production and trade networks influence country-level nourishment. We find that relatively few countries in Europe and North America control the wheat supply chain. The centrality of these countries as opposed to volume of wheat produced or traded, determines their influence in the wheat supply chain network. The parametric component of our analysis confirms that trade, and centrality have significant implications for national levels of nourishment.

For countries with low purchasing power, trade allows improvements in nourishment levels but for countries with very high purchasing power, trade is associated with increased hunger outcomes. The second part of this finding may seem counter intuitive. While one might expect that counties that are central to the global grain trade and have higher purchasing power parity would have greater nourishment, our findings support a growing body of literature that documents how super-exporter countries are so heavily engaged in trade that they may undercut the food security of their own people through extractive economies that aim to achieve a comparative advantage in the economic system over the food system. Our results suggest that extensive exports may contribute to increased delocalization and destabilization of national food systems, and a high degree of risk is borne by Global South countries. Though there is growing research on food supply networks, and food security, our study is unique in tying both aspects empirically, by fitting centrality measures into a predictive model that focusses on hunger outcomes.

Our study has some limitations. First, the SAR analysis includes only 141 countries. Missing undernourishment data, and trade data agglomeration led to over 60 countries, especially from Africa and small island states being dropped from the analysis. Though the results are robust, improved data availability for these regions will also improve the depth and quality of the analysis. Second, grain networks are also embedded within complex economic systems that are tied with various inputs and outputs. Although we consider purchasing power parity and volume of cereals and by-products, there is more complexity in how trade is embedded within larger economic systems that our model does not account for.

While our work points to the importance of centrality in the global wheat network structure and its effect on nourishment outcomes, there are still gaps in our understanding of what strengthens or erodes food systems resilience. Given how important modularity is to systems resilience, future research should integrate measures of local food systems and model how local food system dynamics may affect global food systems. The war in Ukraine also provides an opportunity to assess how food systems, and nutrition is affected, when two of the largest wheat producing nodes are shutdown.
